# Cows visually discriminate and cross-modally recognise familiar and unfamiliar human faces in videos

**DOI:** 10.1371/journal.pone.0329529

**Published:** 2026-05-20

**Authors:** Océane Amichaud, Julie Lemarchand, Fabien Cornilleau, Plotine Jardat, Vitor H. B. Ferreira, Ludovic Calandreau, Léa Lansade

**Affiliations:** INRAE, CNRS, Université de Tours, Centre Val de Loire UMR Physiologie de la Reproduction et des Comportements, Nouzilly, France; University of Vienna: Universitat Wien, AUSTRIA

## Abstract

Social recognition has been studied and demonstrated in many species. In domesticated species, the long evolutionary history shared with humans has led to investigations into their cognitive abilities towards humans, particularly regarding discrimination and recognition of humans. The present study investigated whether cows are capable of visual discrimination and cross-modal recognition of familiar and unfamiliar humans. Thirty-two cows were exposed to two tests: a visual preference test, during which two silent videos were shown simultaneously – each displaying either a familiar or an unfamiliar human face – and a cross-modal test, during which the videos were accompanied by either a congruent or incongruent voice. During the visual preference test, cows looked significantly longer at the video showing the unfamiliar person (p = 0.028). In the cross-modal test, they looked significantly longer at the video that was congruent with the voice being played (p = 0.014). These two results show that cows are able to discriminate between familiar and unfamiliar individuals and form cross-modal representations of these people. Based on these results, future research should explore whether cows can adjust their behaviour depending on the person they are interacting with – a capacity that may reflect their agency in human-animal relationships.

## Introduction

Social recognition is a key mechanism in social animals, as it regulates interactions between individuals and structures the organisation of societies [1]. It can be defined as the ability to categorise conspecifics according to different classes like familiarity, kin, hierarchical status, sex, individual identity etc. [2] and has been demonstrated in many animal species [3]. One of its fundamental prerequisites is the ability to discriminate between individuals [4], which supports the formation and maintenance of social bonds within groups. To achieve this, some species rely primarily on a dominant sensory modality, while others integrate multiple sensory channels such as visual, olfactory, and auditory cues [5].

Among the sensory modalities involved in social recognition, vision plays a central role in many animal species [6–9]. Particularly, facial perception is considered a key component of social recognition, as faces convey important information such as age, sex, and individual identity [10,11]. The visual modality has been widely investigated in domestic species using two-dimensional facial images as stimuli. Several studies have shown that animals can visually discriminate their own species from other species [6,7,12,13], and can also distinguish between familiar and unfamiliar conspecifics based solely on facial cues [10,14–16]. They can also discriminate conspecifics using only auditory or olfactory cues. For instance, dogs and horses can respectively distinguish conspecifics on the basis of their barking or urine samples [17,18]. All of these studies are based on the discrimination of conspecifics using a single sensory modality. Some studies have gone further, investigating whether animals are capable of associating current sensory cues with information previously acquired through different modalities [19]. This ability is known as cross-modal recognition and has been predominantly studied through paradigms matching visual and auditory modalities [19–22]. For example, Proops *et al*. [22] evaluated the ability of horses to individually recognise herd members cross-modally. To do this, they presented horses with a familiar conspecific and then played a vocalisation that had been recorded either from the horse that had just been seen (congruent trial) or from another horse (incongruent trial). Horses’ reaction times and gaze durations differed according to the type of trial, suggesting that horses possess a multimodal representation of familiar individuals.

Domestic species have a long evolutionary history with humans. As humans are an integral part of the environment of domestic animals, it is essential to study how these animals perceive and process human signals in order to achieve a better and more comprehensive understanding of the human-animal relationship. Recent studies have focused on their interspecific socio-cognitive abilities towards us. In particular, researchers have notably examined the ability of domestic mammals to visually discriminate and recognise humans [23]. For instance, sheep can recognise familiar and unfamiliar human faces as in a post-training choice test, they preferred to choose the familiar faces they had learned over unfamiliar ones [24]. Horses can learn to discriminate photographs of unrelated individuals, fraternal and identical twins [25], while dogs recognise their owners’ faces in photographs by approaching them more [26]. Similarly, horses can recognise a photograph of their keeper even if they have not seen them for six months, choosing their keeper’s face over a stranger’s [27]. They can also recognise human faces, choosing the rewarded face at above chance-level, even when they are altered by changes in colour, covered eyes, or different hairstyle [28]. To assess whether individuals can discriminate between two stimuli, visual preference tests are often used. This approach has been employed in various studies, for example in goats to test their ability to discriminate between familiar and unfamiliar human faces [29]. In such tests, the direction of the preference depends on the paradigm, the stimuli, and the species. Looking times may be longer for novel stimuli [16,30], or, conversely, for familiar ones [16,31]. Regardless of the direction of the preference, differences in gaze duration between stimuli suggest that individuals are able to discriminate between them. Another type of test allows us to go beyond simple discrimination: the cross-modal recognition test, which makes it possible to determine whether animals can form cross-modal representations of humans. This paradigm has notably been used in horses and dogs [32–35]. These animals displayed differential looking behaviour depending on the congruency between visual and auditory stimuli, suggesting that they integrate human-related information across sensory modalities.

Socio-cognitive abilities towards humans have been poorly studied in cows [23]. However, dairy cows, in particular, live in close contact with humans from birth, being bottle-fed by humans or milked daily, for example. As mentioned, there is a growing body of evidence suggesting that domestic animals can discriminate and/or recognise human faces, but these abilities have not yet been demonstrated in cows. Given the number of species in which human recognition has been demonstrated, it would be surprising if this were not the case with cattle, thus warranting further investigation. Indeed, cattle are a good model for studying facial discrimination and recognition of familiar and unfamiliar humans. They are social animals and were domesticated 10,500 years ago [36]. They possess good visual acuity and a large visual field (330°) [37]. Moreover, previous studies have shown that cows can discriminate between individual people using multiple visual cues such as body height, colour of clothes and faces; although cows succeed at facial discrimination only when the body is visible and people are presented at the same size and wearing the same clothes [38–40]. One of these studies also reported that cows occasionally discriminated faces alone, but their performance was not stable enough to reliably conclude that they can distinguish people based solely on facial cues [38]. Cows have also been shown to recognise conspecifics in photographs, successfully learning to choose a photograph of a specific individual [41]. From a welfare perspective, a better understanding of their socio-cognitive capacities is therefore essential to improve human-animal interactions and management practices.

The present study uses a preferential looking paradigm to assess cows’ abilities: 1) to visually discriminate between familiar and unfamiliar human faces (visual preference tests) and 2) to cross-modally recognise humans, by associating their voice with their face (cross-modal tests). For the visual preference tests, the hypothesis was that cows’ looking durations would differ between familiar and unfamiliar individuals. For the cross-modal tests, our hypothesis was that the animals would be able to associate the faces of individuals with their voices. This would likely result in varying gaze durations depending on the congruence between the observed face and the heard voice. At this stage, it was difficult to predict the direction of these variations, since this is one of the first studies to include cows in such a paradigm, and the literature suggests that the direction of the effect can vary both within and between species. For instance, horses generally look longer at faces that are incongruent with the voice [32,33,42,43], while dogs generally do the opposite [44,45]. We also hypothesised that cows would experience different emotional arousal in response to familiar and unfamiliar stimuli, which could result in differences in heart rate variation in response to the voices in the cross-modal tests.

## Materials and methods

### Ethics statement

All methods were carried out in accordance with the relevant guidelines and regulations, and in compliance with the Declaration of Helsinki. The volunteers filmed in this study were colleagues from our INRAE unit and were informed about the aims and methods of the study prior to their participation. All provided written consent for the recording and use of their images. This study underwent an ethical self-evaluation following the guidelines of the INRAE Ethics of Projects Committee, and the experimental protocol was approved by the Val de Loire Ethical Committee (CEEA VdL, Nouzilly, France, authorization number CE19–2025-2502-1).

### Subjects and housing

The study involved 34 Prim’ Holstein cows (*Bos taurus taurus*) aged 21.6 ± 15.3 months (mean ± SD; details in S2 Table) reared at the Experimental Unit PAO (UEPAO, 37,380 Nouzilly, France, https://doi.org/10.15454/1.5573896321728955E12), INRAE. This sample size was chosen because it is similar to that used in studies investigating comparable cognitive abilities in other species [5,15]. These cows were housed in groups in indoor stalls enriched with automatic brushes and bedded with straw. Water was available ad libitum. They had been handled daily for feeding and care by four caretakers since birth, but they may also occasionally have encountered other individuals, such as students or colleagues visiting the farm. All cows used in this study had no prior exposure to similar experimental procedures.

### Experimental setup

**Fig 1:**
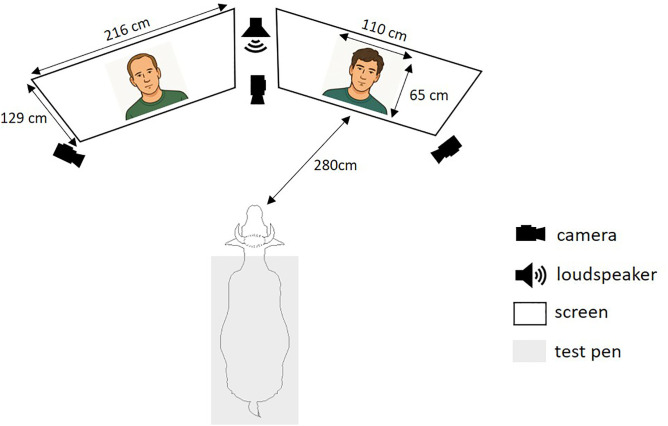
Experimental setup for visual preference and cross-modal tests. The cow was positioned centrally between two screens. Each screen showed a video of a person’s face: one familiar and one unfamiliar to the cow. During cross-modal tests, a speaker placed between the screens played the voice of one of the two individuals. Cameras recorded the cow’s behavioural responses throughout the test.

The experiment took place in a barn housing cows not included in the study, adjacent to the home barn where the participating cows were kept. Before each test session, the experimenters ensured that, to the ear, no external sounds (human voices or farm machinery) were audible from the barn. The tested cow was placed in a test pen (l.159 x w.90 x h.192 cm; Fig 1). The videos were projected onto two projection screens. Each test was filmed by three cameras (FDR-AX43A, Sony, Japan), one placed between the screens and the other two on either sides of the screens (Fig 1). The cows were fitted with a heart rate sensor belt (Polar Equine, Polar, Finland). The sensor was linked via Bluetooth to the Polar Beat smartphone application to display and record heart rate in real time.

### Video preparation

Eight adult men (approximately 30–60 years old), including four familiar caretakers who provided daily care to the cows and four unfamiliar colleagues the cows had never seen before, were filmed prior to the experiment. We selected only men to avoid discrimination based on sex. They were recorded in 4K by camera (FDR-AX43A, Sony, Japan) under similar conditions, in the same room, at the same location within the room, with artificial light only and with the same framing (centred in the image and shoulders visible, at 30 cm from the camera). All the men were recorded looking at the camera lens and saying the following sentences with a neutral expression: « La réunion commence bientôt, je vais chercher mes affaires. Le chat monte dans l’arbre. », meaning « The meeting starts soon, I am going to get my things. The cat is climbing the tree. ». These sentences were chosen because they contain words that cows are not used to hearing. Using the Audacity software (v. 3.7.1, https://www.audacityteam.org/), the RMS (Root Mean Square), which measures the average intensity of the men’s speech, was calculated from the audio of the recordings. The sound of each video was adjusted to match the mean RMS value across all eight men (−25 dB).

### Tests

**Fig 2:**
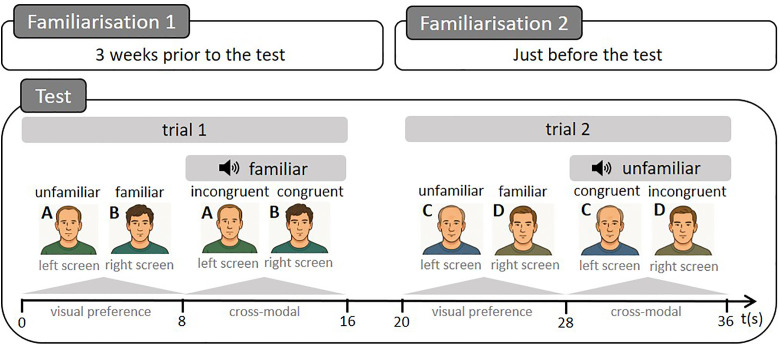
Experimental procedure. Following the familiarisation phases, the cows were subjected to a test phase consisting of two trials. Each trial included a visual preference test lasting 8 seconds, during which two faces were simultaneously presented on the two screens: one familiar face and one unfamiliar face. This test was followed by a cross-modal test, also lasting 8 seconds, during which the voice of one of the two individuals was played. The video was considered congruent when the displayed face corresponded to the played voice, and incongruent when the voice did not match the displayed face. A, B, C and D represent different men.

After the familiarisation phases (see Fig 2 and S1 Appendix for details), the animals underwent two successive tests: the visual preference test, immediately followed by the cross-modal test (Fig 2). During the test sessions, two previously prepared portrait videos were shown simultaneously (Fig 1): a video of a familiar man and a video of an unfamiliar man. This sequence was repeated a second time with different stimuli (faces and voice) after a 4-second interval during which black screens were projected (Fig 2).

#### Visual preference test.

The visual preference test lasted 8 seconds (Fig 2). Two muted videos, one showing a familiar face and the other an unfamiliar face, were presented simultaneously (Fig 1). Familiar and unfamiliar faces were randomly selected from a pool of four familiar and four unfamiliar individuals. The presentation side of the familiar and unfamiliar faces was also randomly assigned and varied between individuals and repetitions.

#### Cross-modal test.

mmediately after the visual preference test, the cows were subjected to a cross-modal recognition test for another 8 seconds (Fig 2). In addition to the same two videos presented during the visual preference test, an audio recording of a voice corresponding to either the familiar or the unfamiliar individual was broadcast by a loudspeaker (Megaboom 3, Ultimate Ears, United States; Fig 1) placed between the two screens and facing the cow. The sound was slightly offset from the image to prevent the cow from associating the voice she heard with the mouth movements of one of the people shown in the video. These voices were broadcast with an approximate intensity of 70 dB from where the cows’ head was, because it corresponds approximately to the intensity perceived when real people are talking. The presentation side of the congruent and incongruent videos, as well as the familiarity of the voice, were also randomly assigned and varied between individuals and repetitions.

### Behavioural and physiological analyses

Videos of the tests were analysed with the BORIS software [46] by the same coder. The screens were not visible on the cameras so that the coder did not know which side the familiar person and the congruent video appeared on. The time spent looking at each screen (right or left from the animal’s point of view) was quantified. The cow was considered to be looking at the right screen when her left eye was not fully visible to the right camera; conversely, she was considered to be looking at the left screen when her right eye was not fully visible to the left camera (Fig 1). For each cow, depending on the test and on the repetition, we obtained the total time spent watching the familiar and unfamiliar person and the time spent watching the congruent and incongruent video. First gaze duration was also measured for each stimulus (familiar/unfamiliar and congruent/incongruent). Both variables are commonly considered in similar tests [22,32,42], notably because significant effects can be observed only on one of these two variables [22]. Twenty percent of the videos (7 individuals) were analysed again by a second coder to assess scoring reliability. Interclass correlation coefficients (ICC) and their 95% confidence intervals were calculated and interpreted according to Koo and Li’s method [47], showing good reliability for total gaze duration (ICC = 0.809[0.695; 0.884]) and moderate reliability for first gaze duration (ICC = 0.636 [0.404; 0.791]).

Heart rate (HR) data were extracted via Polar Flow. For each cow, we removed HR values that were below 40 and above 180 bpm, as these values were considered artefactual [48]. For the cross-modal test of each repetition, mean HR and HR variation (difference between the last 3 and first 3 seconds) were calculated for each cow.

### Statistical analyses

All statistical analyses were performed using R version 4.4.2 [49] and the results are summarised in Table 1. All the figures presented in the results section were produced using the package *ggplot2* [50]. The significance threshold was fixed at p ≤ 0.05.

**Table 1:** Summary of the results of the generalised mixed models. Gaze durations were analysed with familiarity of the person (visual preference tests) and video congruence (cross-modal tests). Heart rate (HR) measures were analysed with the familiarity of the voice. Significant results are shown in bold.

Test	Response variable	Explanatory variable	Estimate	95% CI	χ²	Z	p-value
Visual preference	First gaze duration	Familiarity of the person	0.520	0.110; 0.931	6.163	2.483	**0.013**
	Total gaze duration	Familiarity of the person	0.438	0.047;0.828	4.817	2.195	**0.028**
Cross-modal	First gaze duration	Congruency of the video	−0.712	−1.162; −0.263	9.661	−3.108	**0.002**
	Total gaze duration	Congruency of the video	−0.581	−1.044; −0.117	6.034	−2.456	**0.014**
	Mean HR	Familiarity of the voice	2.479	−2.333; 7.291	1.020	1.010	0.313
	HR variation	Familiarity of the voice	−0.597	−2.822; 1.629	0.276	−0.525	0.599

For the variables total gaze duration and first gaze duration, we excluded, for each cow, trials in which the animal looked at only one of the two screens, as such cases cannot be used to assess a visual preference. After applying the exclusion criteria, data from 22 cows were retained for the visual preference analysis, and from 17 cows for the cross-modal analysis, out of an initial sample of 32 cows. To determine whether the cows preferred a person and whether they were sensitive to the congruence between voices and faces, the total gaze duration and first gaze duration for each screen were analysed according to the person presented (for the visual preference tests) and according to video congruence (for the cross-modal tests). For this analysis we used generalised mixed effects models (GLMMs) from the *glmmTMB* package [51] with gaussian distributions. For each of the two response variables, two models were constructed (one per test): one to analyse the effect of the familiarity of the person presented and the other to analyse the effect of the congruence of the video. The identity of the cow and trial number were added as random effects to account for individual variations and to control for potential heterogeneity between trials. Distributions, homoscedasticity of the residuals and the homogeneity of the variances were verified for the model fitting with the *DHARMa* package [52]. Response variables were log-transformed to improve model fit. All models were compared to their respective null models and were found to differ significantly (S1-S2 Tables). Additionally, in the cross-modal condition, the interaction between congruence and voice familiarity was tested for both first gaze duration and total gaze duration, and did not reach statistical significance (GLMMs; respectively: χ^2^ = 1.739; df = 1; Z = −1.319; p = 0.187; χ^2^ = 2.851; df = 1; Z = −1.689; p = 0.093).To determine whether cows reacted to the voice they heard, HR variation and mean HR were analysed according to the voice broadcast during cross-modal tests (familiar vs unfamiliar voice). For this analysis, we used GLMMs from the *glmmTMB* package [51] with gaussian distributions. The identity of the cow was added as a random effect to account for individual variations. Distributions, homoscedasticity of the residuals and homogeneity of the variances were verified for the model fitting with the *DHARMa* package [52].

## Results

### Visual preference tests

**Fig 3:**
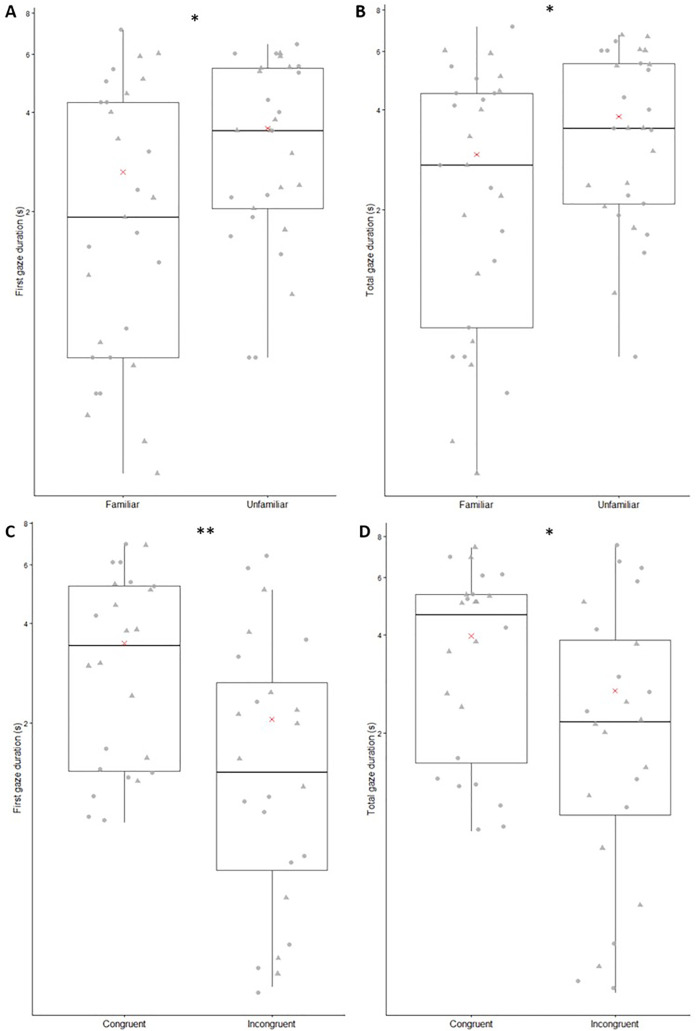
First gaze (A, C) and total gaze (B, D) durations. Figures A and B show results from the visual preference test, according to the familiarity of the human face. Figures C and D show results from the cross-modal test, according to the congruence of the video. Boxplots indicate the median (central line) and the first and third quartiles (box limits). Red crosses represent means. Circular points represent individual values for trial 1, and triangular points represent individual values for trial 2. *: p ≤ 0.05, **: p ≤ 0.01, GLMMs.

In the visual preference tests, the first gaze duration and the total gaze duration were significantly longer towards the unfamiliar person (GLMMs; respectively: χ^2^ = 6.163; df = 1; Z = 2.483; p = 0.013; Fig 3A; χ^2^ = 4.817; df = 1; Z = 2.195; p = 0.028; Fig 3B).

### Cross-modal tests

First gaze duration and total gaze duration were significantly longer towards the congruent video (GLMMs; respectively: χ^2^ = 9.661; df = 1; Z = −3.108; p = 0.002; Fig 3C; χ^2^ = 6.034; df = 1; Z = −2.456; p = 0.014; Fig 3D).

The mean HR did not differ according to the voice broadcast (familiar voice: 84.84 ± 9.51 bpm; unfamiliar voice: 87.59 ± 11.51 bpm; GLMM; χ^2^ = 1.020; df = 1; Z = 1.010; p = 0.313). The HR variation between the first 3 and last 3 seconds did not differ either (familiar voice: 1.02 ± 3.72 bpm; unfamiliar voice: 0.38 ± 5.08 bpm; GLMM; χ^2^ = 0.276; df = 1; Z = −0.525; p = 0.599).

## Discussion

The present study investigated whether cows are capable of visual discrimination and cross-modal recognition of familiar and unfamiliar humans. During the visual preference tests, cows looked significantly longer at the unfamiliar person, suggesting that they are able to discriminate between familiar and unfamiliar individuals using only a video of their faces as a cue. During the cross-modal tests, cows looked significantly longer at the face that matched the voice, indicating that they are able to associate familiar and unfamiliar voices with the corresponding face. However, they did not seem to show a physiological difference according to the voice heard, as the HR variables did not differ according to the familiarity of the voice.

### Visual preference tests

Using visual preference tests, we observed a difference, both in the first gaze duration and in the total gaze duration, between the gazes directed at the familiar person and the unfamiliar person. More time spent watching a video indicates a preference for a given stimulus and suggests that the animal discriminates between the stimuli presented [53]. The observed results support the view that cows can categorise human faces according to familiarity. Thus, the capacity for differentiating human faces based on visual cues alone found in other domestic species [16,27] could extend to cows. Moreover, our results show that cows spent more time looking at the unfamiliar human, both in terms of first gaze duration and total gaze duration. Paradoxically, when exposed to photos of conspecifics’ heads, heifers spent more time looking at photos of familiar heifers than at photos of unfamiliar heifers [10]. The preference for looking at familiar or unfamiliar individuals could depend on whether the subject is a conspecific or a human. A similar observation has been made in dogs: using a visual preference paradigm, Racca *et al.* [16] showed that dogs preferred to look at unfamiliar faces when presented with human faces, but preferred familiar faces when presented with conspecific faces. Unfamiliar human faces, due to their novelty, may be perceived as potentially threatening, thereby eliciting heightened vigilance and an increased allocation of attention towards the stimulus.

### Cross-modal tests

Our results indicate that cows’ abilities to perceive and process human signals seem to go beyond unimodal processing. In line with our hypothesis, cows watched the videos for varying lengths of time, depending on their congruence with the voice they heard, regardless of the familiarity of the voice. Cows’ ability to differentiate between familiar and unfamiliar humans in the videos, based on their congruence with the voice heard simultaneously, suggests that cows are capable of multimodal processing of human signals beyond facial or visual cues. More specifically, cows watched the congruent video for longer, i.e., the video presenting the person whose voice was being broadcast, for both first and total gaze durations. This longer gaze towards the congruent stimulus is in line with other studies using a cross-modal paradigm [5,35]. For instance, Proops and McComb [5] tested whether horses were capable of individual recognition of familiar human handlers and showed that horses spent more time looking at the congruent stimulus. In contrast, studies investigating horses’ cross-modal representation of children and adults or of human facial emotional expressions, as well as studies on dogs’ and cats’ ability to form cross-modal representations of individual humans, have shown that these animals spend more time looking (overall or for first gaze) at the stimulus that was incongruent with the sound [32,33,42,43,54,55]. These differences may be explained by variations in the species studied or in the experimental conditions, such as differences in the emotions or stress levels elicited by the presented stimuli.

In our study, as indicated by cows’ heart rate responses, familiar and unfamiliar auditory stimuli did not seem to induce different levels of emotional arousal, contrary to what we had predicted. In horses, a study also using a cross-modal paradigm, reported no effect of men’s versus women’s voices on HR measures and observed, as we did, more attention toward congruent stimuli [35]. Conversely, Jardat *et al*. [33], who investigated cross-modal representations in horses in both adults and children, reported increased heart rate when hearing children’s voices and longer looking durations at the incongruent stimuli, possibly reflecting stress or surprise, as the horses had never encountered children before. In our study, although the cows tested had never seen or heard the unfamiliar people presented, prior exposure to other new humans, including unfamiliar male voices, may have reduced their physiological reactivity to unfamiliar voices and could explain the absence of an increased heart rate response to these voices during the test. Other hypotheses may also account for the absence of variation in heart rate in response to familiar and unfamiliar voices. Firstly, it is possible that cows discriminate between familiar and unfamiliar voices, but this does not necessarily imply that such discrimination is accompanied by a significant difference in emotional responses, particularly in terms of arousal level. It has been shown that goats are able to discriminate the emotional valence of human voices, however, this behavioural response was not accompanied by a significant physiological change [56]. Secondly, heart rate analyses were conducted during cross-modal tests, which involved relatively short durations of voice emission (eight seconds). This period may be too short to show any variation in heart rate. For instance, in a cross-modal test, Jardat *et al.* [33] showed that the horses’ heart rate increased when they heard a child’s voice, but their repetitions lasted twice as long (16 seconds). Moreover, in our study, the voice was not presented in isolation but simultaneously with two visual stimuli, which may have further reduced the likelihood of eliciting a clear physiological response to the sound. In such a context, where the stimulus does not elicit a strong emotional reaction, cows may be more inclined to direct their attention towards the congruent video, as observed in the present study. Other physiological measures commonly used to assess emotional responses, such as ocular temperature [57–59] or heart rate variability [60–62], could have been combined with our own measures to further support or refute the absence of differences in emotional responses as a function of the familiarity of the presented voice.

This study has several notable strengths and offers novel insights into bovine socio-cognitive abilities. To our knowledge, it is the first study to apply a cross-modal paradigm in cows, widely used to investigate heterospecific socio-cognitive abilities in other domestic species [33,34,43,54]. Human faces were presented exclusively as two-dimensional videos, an artificial format lacking depth cues and additional information such as scent, voice, body shape, posture, or gait. While this limitation means the stimuli may not fully replicate real-life interactions, the use of videos as standardised 2D stimuli also offers key advantages: it improves reproducibility and comparability of results and incorporates an additional sensory modality, namely the auditory channel, which is not possible with photographs. Until now, studies on cow socio-cognitive abilities towards humans employed either real humans [38–40,63] or photographs [40], making our approach particularly innovative. Finally, this research makes a significant contribution to our understanding of socio-cognitive abilities in cows, a species for which few studies have explored human-directed socio-cognitive skills.

Our findings suggest that cows are capable of processing human cues and that they do not perceive all humans as a single, undifferentiated category, but are instead capable of distinguishing and recognising individuals they have previously met. Our results also indicate that cows are capable of integrating multiple sensory cues, reflecting a higher level of cognitive processing than that required for unimodal recognition, for example. Indeed, the ability to combine information from different sensory modalities suggests that cows form multisensory representations of individual humans. Based on these observations, it is therefore advisable to maintain a consistent caregiving staff to strengthen the human-animal relationship and to ensure that caregivers adopt coherent behaviours across different sensory channels. This study adds to a growing body of research showing that domesticated mammals develop complex socio-cognitive abilities in their interactions with humans [23]. Humans are part of their environment, particularly by providing them with daily care (e.g., by feeding or petting them) and animal welfare depends directly on the way in which an animal is able to perceive, interpret and analyse its environment. The recent definition of positive welfare emphasises the importance of experiencing mainly positive mental states, notably through the opportunity to make choices [64]. For animals, the possibility of making choices can have a direct positive impact on their emotional state by giving them a feeling of control over interactions with their environment [65]. In the context of the human-animal relationship, this is illustrated, for example, by giving the animal a choice as to when and how to interact [66]. In this way, the socio-cognitive abilities mentioned above may have an adaptive value and enable animals to adjust their behaviour according to the person’s profile, thereby supporting animals’ active role in shaping their social environment within a positive welfare framework.

## Conclusion

In this study, using visual preference and cross-modal tests, we showed that cows are able to process human faces presented in 2D on videos and to associate familiar and unfamiliar faces with the corresponding voices by integrating multiple sensory modalities. However, this is not accompanied by significant differences in cows’ physiological responses depending on the person’s familiarity. Such cognitive abilities highlight the complexity of human perception in domestic animals, as discussed in the review by Jardat and Lansade [23]. In cows, this innovative experimental design provides a promising tool for investigating a wider range of cognitive abilities in this species, notably their ability to recognise individual humans and to develop preferential interactions. A better understanding of how cows perceive and differentiate humans could help inform husbandry practices that incorporate human–animal interactions aligned with their cognitive abilities, in order to provide them with greater opportunities for choice and initiative in their relationship with humans – thereby reinforcing their agency, a key component of positive welfare [64,67].

## Supporting information

S1 Appendix Familiarisation procedure. (DOCX)

S1 Table Identity numbers and ages (in months) of the cows included in the study. (DOCX)

S2 Table Comparison of statistical models and corresponding null models. Models in bold indicate those identified by the ANOVA as significantly different from their respective null models. These selected models correspond to those previously specified and retained (see Statistical Analysis), and are therefore reported in the Results section. Statistical significance was set at p ≤ 0.05.
(DOCX)
