# Supplementary material for: Cows visually discriminate and cross-modally recognise familiar and unfamiliar human faces in videos
Source: PLoS One. 2026 May 20;21(5):e0329529. doi: 10.1371/journal.pone.0329529 (PMC13189301; doi:10.1371/journal.pone.0329529)
Supplement: S1 Appendix — (DOCX) [file pone.0329529.s001.docx]

**S1 Appendix. Familiarisation procedure.**

A familiarisation session, conducted prior to testing days, was used to familiarise the cows with the test environment. They were familiarised with crossing the outdoor courtyard to reach the testing stall accompanied by caretakers. In the test pen, they were also familiarised with being fitted with the heart rate monitor belt. Once the cow was in position and equipped, she had to stay five minutes in the experimental setup or to maintain a heart rate below 95 beats per minute (bpm) for two consecutive minutes before she was returned to her home pen. This threshold of 95 bpm was chosen because it is higher than the basal heart rate of young and adult bovids and has been exceeded following acute stress in several studies [45,46]. A second familiarisation session, identical to the first, was conducted for each cow immediately prior to testing. If the cow maintained a heart rate below 95 bpm for two consecutive minutes, the test phase began immediately (28 cows). If the threshold was not reached within five minutes, the test was postponed to the following day, with another familiarisation session carried out beforehand (6 cows). Of the remaining six cows, four succeeded on their second attempt. The final two cows did not meet the criterion even after the second session and were excluded from testing, as the procedure was considered too stressful for them. In total, 32 cows completed the test phase, and analyses were conducted on these 32 individuals.
