# Supplementary material for: Cows visually discriminate and cross-modally recognise familiar and unfamiliar human faces in videos
Source: PLoS One. 2026 May 20;21(5):e0329529. doi: 10.1371/journal.pone.0329529 (PMC13189301; doi:10.1371/journal.pone.0329529)
Supplement: S1 Table — (DOCX) [file pone.0329529.s002.docx]

**S1 Table. Identity numbers and ages (in months) of the cows included in the study.**

| Cow identity number | Age in months |
| --- | --- |
| 2000 | 60 |
| 2002 | 49 |
| 2012 | 59 |
| 2031 | 51 |
| 2104 | 44 |
| 2203 | 34 |
| 2216 | 27 |
| 2221 | 27 |
| 2226 | 26 |
| 2227 | 26 |
| 2228 | 26 |
| 2229 | 26 |
| 2318 | 15 |
| 2320 | 15 |
| 2326 | 15 |
| 2328 | 15 |
| 2331 | 15 |
| 2333 | 14 |
| 2335 | 14 |
| 2337 | 13 |
| 2341 | 13 |
| 2342 | 11 |
| 2343 | 11 |
| 2344 | 10 |
| 2345 | 10 |
| 2347 | 10 |
| 2348 | 10 |
| 2401 | 10 |
| 2402 | 10 |
| 2405 | 9 |
| 2407 | 9 |
| 2408 | 9 |
