# Supplementary material for: Cows visually discriminate and cross-modally recognise familiar and unfamiliar human faces in videos
Source: PLoS One. 2026 May 20;21(5):e0329529. doi: 10.1371/journal.pone.0329529 (PMC13189301; doi:10.1371/journal.pone.0329529)
Supplement: S2 Table — Models in bold indicate those identified by the ANOVA as significantly different from their respective null models. These selected models correspond to those previously specified and retained (see Statistical Analysis), and are therefore reported in the Results section. Statistical significance was set at p ≤ 0.05. (DOCX) [file pone.0329529.s003.docx]

**S2 Table.** **Comparison of statistical models and corresponding null models.** Models in bold indicate those identified by the ANOVA as significantly different from their respective null models. These selected models correspond to those previously specified and retained (see Statistical Analysis), and are therefore reported in the Results section. Statistical significance was set at p ≤ 0.05.

| **Test** | **Response variable (y)** | **Model type** | **Family** | **Formula** | **χ²** | **DF** | **p value** | **AIC** | **BIC** |
| --- | --- | --- | --- | --- | --- | --- | --- | --- | --- |
| Visual preference | Total gaze duration | GLMM | gaussian | y ~ 1 |  |  |  | 145.28 | 153.52 |
|  |  |  |  | **y ~ familiarity** | **4.627** | **1** | **0.031** | **142.65** | **152.96** |
|  | First gaze duration | GLMM | gaussian | y ~ 1 |  |  |  | 152.30 | 160.54 |
|  |  |  |  | **y ~ familiarity** | **5.857** | **1** | **0.016** | **148.45** | **158.75** |
| Cross-modal | Total gaze duration | GLMM | gaussian | y ~ 1 |  |  |  | 130.72 | 138.20 |
|  |  |  |  | **y ~ congruence** | **5.683** | **1** | **0.017** | **127.03** | **136.39** |
|  | First gaze duration | GLMM | gaussian | y ~ 1 |  |  |  | 130.87 | 138.35 |
|  |  |  |  | **y ~ congruence** | **8.802** | **1** | **0.003** | **124.07** | **133.42** |
